# Effects of Reinforcement Corrosion and Sustained Load on Mechanical Behavior of Reinforced Concrete Columns

**DOI:** 10.3390/ma15103590

**Published:** 2022-05-18

**Authors:** Qiang Li, Zheng Dong, Qi He, Chuanqing Fu, Xianyu Jin

**Affiliations:** 1 Key Laboratory for Technology in Rural Water Management of Zhejiang Province, Zhejiang University of Water Resources and Electric Power, Hangzhou 310018, China; liqiangtzh@126.com; 2 College of Civil Engineering, Zhejiang University of Technology, Hangzhou 310023, China; chqfu@zjut.edu.cn; 3 College of Civil Engineering and Architecture, Zhejiang Tongji Vocational College of Science and Technology, Hangzhou 311231, China; heqiqihe@yeah.net; 4 College of Civil Engineering and Architecture, Zhejiang University, Hangzhou 310034, China; xianyuj@126.com

**Keywords:** reinforcement corrosion, accelerated corrosion test, reinforced concrete, sustained load

## Abstract

The effects of reinforcement corrosion and sustained axial load on mechanical performance of reinforced concrete (RC) columns were investigated in the present study. Three different degrees of reinforcement corrosion were achieved by controlling the durations of accelerated corrosion test (i.e., 16 days, 31 days, and 63 days). Three levels of sustained axial load (i.e., 0%, 30%, and 60% of the ultimate bearing capacity) were concentrically applied on column specimens. The impressing current and the sustained load were applied on column specimens simultaneously, mimicking the degradation of RC columns in real structures. Results indicated that transverse stirrups yielded higher corrosion degree than that of the longitudinal rebar under identical duration of accelerated corrosion test. The application of sustained axial load improved the performance of corroded RC columns in terms of the reinforcement corrosion, the ultimate axial load, as well as the stiffness. Additionally, more longitudinal cracks along the main rebar were exhibited for column specimens subjected to sustained axial load. For both loaded and unloaded column specimens, corrosion of reinforcing steels exacerbated the mechanical deterioration of RC columns, lowering the ultimate load carrying capacity and the axial deformation compared to the uncorroded columns.

## 1. Introduction

Reinforced concrete (RC) structures are subjected to various loading conditions and attack of aggressive ions in severe environments [1,2,3,4,5,6]. Reinforcement corrosion, as one of the main causes for the deterioration of RC structures [4,7,8,9,10,11], affects the mechanical behavior of RC members in several ways such as the loss of cross-sectional area of corroding steel [12,13,14], the cracking and spalling of concrete cover due to the expansive corrosion products [10,15,16,17,18], the reduction of steel-to-concrete bond strength [19,20,21,22], etc.

With regard to the effects of reinforcement corrosion on the performance of RC columns which are the main members in RC structures to resist axial compressive loads, generally, corroded reinforcing steels suffer loss of strength and ductility [23,24]. Additionally, the cracking of concrete cover due to reinforcement corrosion results in the reduction of concrete cross section. On the other hand, the corrosion of transverse stirrups decreases the confinement to concrete. The properties of RC columns are thereby reduced, including the ultimate load carrying capacity, stiffness, ductility [3,5,23,24,25,26,27], etc.

To evaluate the load carrying capacity of corroded RC columns, the method presented by Tapan considers the reduction in the yield strength of corroded reinforcement, the loss of bond, the buckling of corroded reinforcement under compression, and the reduction in concrete cross section [23,24]. In addition, the eccentricity of the external load as well as the location of corroded reinforcement (i.e., in compression zone or tension zone) affects the degradation of load carrying capacity for RC columns as well [1,3,28,29]. Nevertheless, in most of the studies mentioned above, the influences of corrosion and sustained load are not considered simultaneously. The corrosion test of reinforcement is generally followed by the mechanical strength test [3,30,31,32], while in real structures, RC members are subjected to simultaneous reinforcement corrosion and sustained loads [1,3,31,33,34,35,36,37]. Reinforcing steels in RC columns subjected to various loading conditions yielded different corrosion activities [38,39,40], which may in turn affect their strength and the amount of expansive corrosion products. Additionally, the sustained loads alter the porosity of concrete matrix [41]. Therefore, it is imperative to investigate the performance of RC columns under simultaneous reinforcement corrosion and sustained loading conditions.

The present study aimed to investigate the mechanical behavior of RC columns under simultaneous reinforcement corrosion and sustained axial load. Three different degrees of reinforcement corrosion and three loading levels of sustained concentric axial load (i.e., 0%, 30%, and 60% of ultimate capacity) were designed. Different degrees of reinforcement corrosion were obtained by accelerated corrosion technique (i.e., galvanic method). The effect of sustained axial load on the reinforcement corrosion, the cracking patterns, the failure modes of RC columns, as well as the load–deformation responses of RC columns were analyzed.

## 2. Materials and Methods

**Figure 1:**
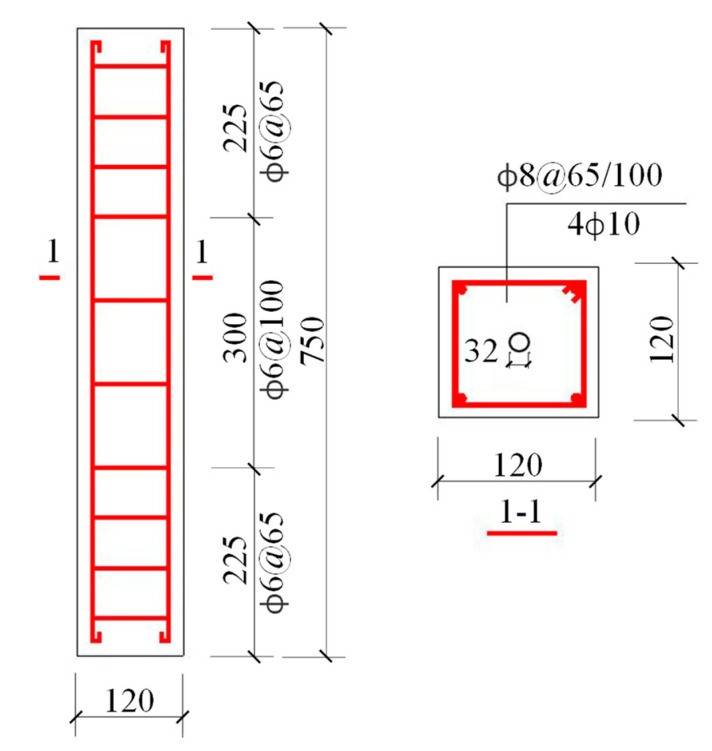
Details of RC column specimens (unit: mm).

A total of 10 RC column specimens were prepared. Table 1 lists the mixture proportion of the specimens. The dimensions of RC columns were 120 mm × 120 mm × 750 mm, as illustrated in Figure 1. A hole with a diameter of 32 mm was designed at the center of the column for the sake of prestressing bar. The cover depth of the column specimens was 15 mm. Hot rolled plain bars (HPB235) were employed as the longitudinal rebar and transverse stirrups in column specimens. The diameters of longitudinal rebar and transverse stirrups were 10 mm and 6 mm respectively, the mechanical properties of which have been reported in Ref. [1]. Additionally, three concrete cubes with the dimensions of 100 mm × 100 mm × 100 mm were cast to measure the average 28-day compressive strength, which was 27.93 MPa.

To investigate the effects of steel corrosion and sustained axial loads on mechanical performance of RC columns, different durations of accelerated corrosion test and various levels of sustained load were designed, as shown in Table 2.

**Figure 2:**
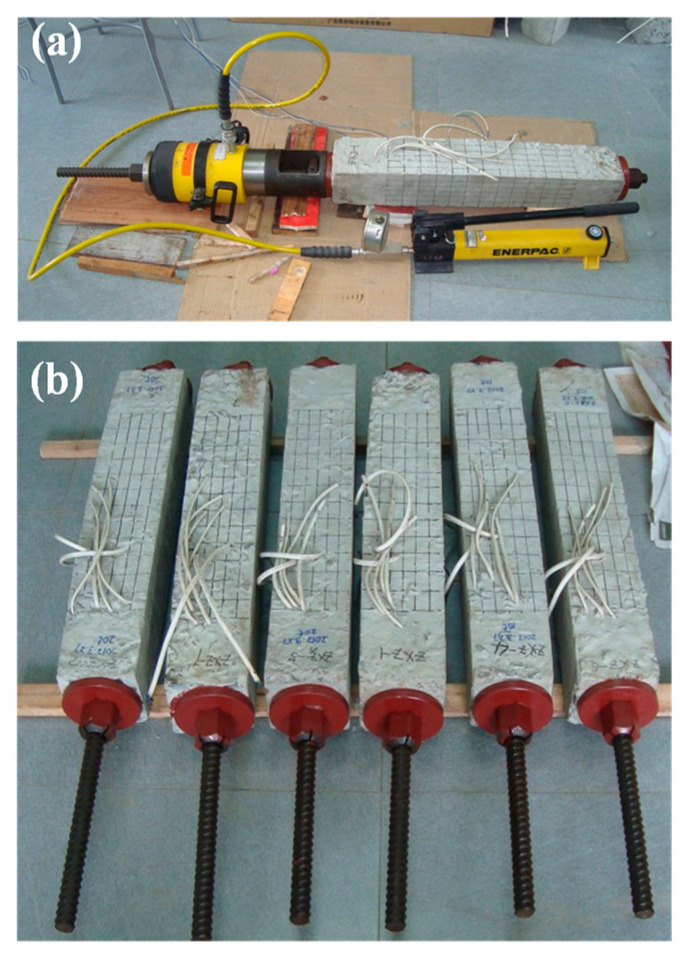
(**a**) Prestressing process and (**b**) preloaded column specimens.

In terms of the application of sustained load, as Figure 2a demonstrated, a self-balancing prestressing method was used [1]. Pressure was generated from the oil pump. The screw-thread steel bar which was positioned through the hole was then tensioned, which in turn exerted compression on the column specimens, as shown in Figure 2b. Periodic examinations were conducted to compensate for the possible stress relaxation, maintaining the level of sustained load.

**Figure 3:**
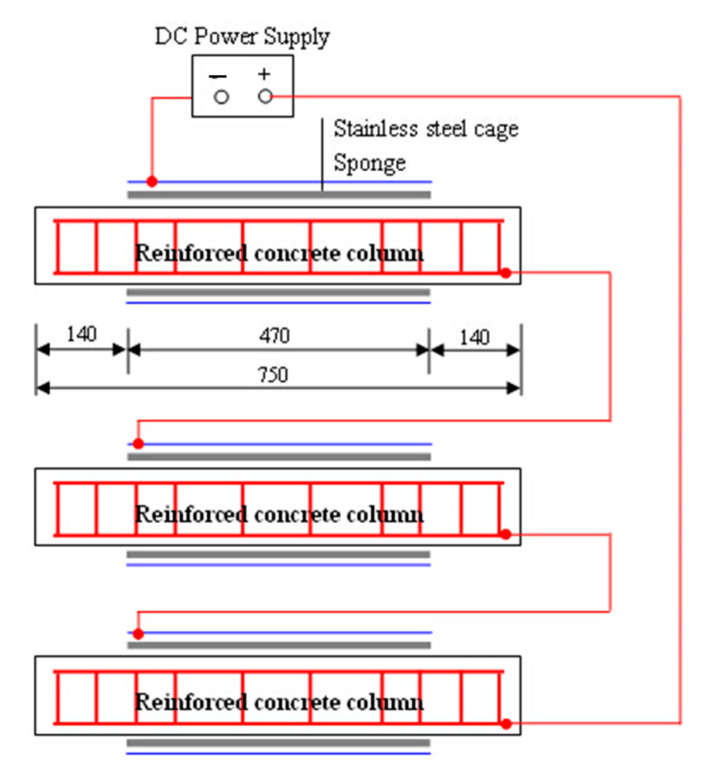
Schematic illustration of accelerated corrosion test setup.

The galvanic method was employed to accelerated the corrosion propagation of reinforcements in columns, as schematically illustrated in Figure 3. To prevent the damage of the anchoring members, epoxy resin was used to cover the ends with a length of 140 mm, leaving an exposed length of 470 mm, as shown in Figure 3. A sponge which soaked up 5 wt.% NaCl solution embraced the exposed length of each column specimen, keeping the concrete wet [42,43]. A stainless-steel mesh was then used to surround the sponge for each specimen. To reduce the loss of moisture in the sponge, the stainless-steel mesh was wrapped by a plastic sheet. During the accelerated corrosion test, the embedded steels including longitudinal rebar and transverse stirrups were connected to the anode. The stainless-steel mesh was connected to the cathode. The impressed current density was 200 μA/cm^2^. The durations of impressing current were designed to be 16 days, 31 days, and 63 days, as listed in Table 2. It should be emphasized that, for specimens subjected to both sustained load and reinforcement corrosion, these two processes were performed simultaneously.

After the accelerated corrosion test, the distribution of corrosion-induced cracks on the surfaces of column specimens was copied by putting a piece of transparent plastic paper with 20 mm × 20 mm grids on the surfaces of columns. The crack width was measured using a crack width gauge.

**Figure 4:**
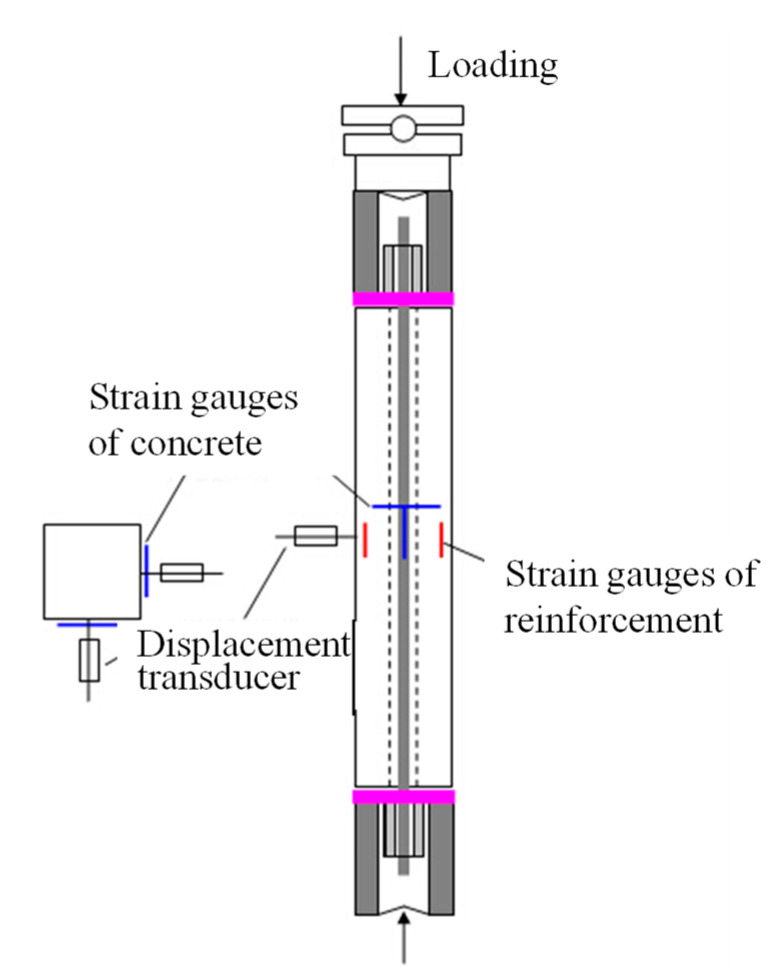
Configuration of loading test.

Afterwards, loading tests were carried out by loading the column specimens using a universal testing machine. The preloaded RC columns were concentrically loaded at the same location of the prestress, as shown in Figure 4. For each column specimen, two strain gauges were arranged on the midspan of two adjacent faces of column, measuring the axial strain and the lateral strain of column specimen. Additionally, two displacement transducers were employed to measure the lateral deformation of column, as depicted in Figure 4. The procedures of the loading test have been reported previously [1]. During the loading tests, the values of axial load, lateral deflection, and axial and lateral strains of RC columns were recorded.

To calculate the mass loss of corroded reinforcement, the longitudinal rebar and transverse stirrups were weighed before the application of sustained load and the impressing current, and after the loading tests, respectively. Prior to the preparation of RC column specimens, all the reinforcing steels were cleaned and weighed. After the loading tests, the corroded reinforcing steels were carefully removed from the broken column specimens. The corroded reinforcing steels were then cleaned according to the standard ASTM G1-03 [44]. An identical cleaning process was conducted on non-corroded control steels. The extent of mass loss caused by cleaning was found to be negligible. The corrosion of steel bar induced by impressing current (i.e., galvanic method) was relatively uniform, especially with a high value of impressed current density [45]. In this regard, the degree of reinforcement corrosion (i.e., the mass loss ratio) was calculated by Equation (1).

(1) $$∆m=\frac{m_{0}-m_{i}}{m_{0}},$$

where *m*_0_ is the mass of non-corroded steel and *m_i_* is the mass of corroded steel.

## 3. Results and Discussion

### 3.1. Degree of Reinforcement Corrosion

**Figure 5:**
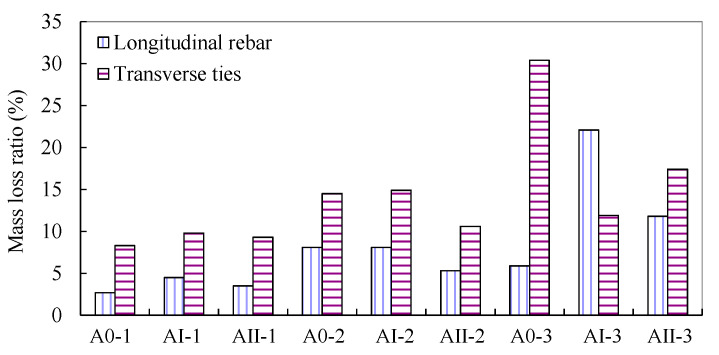
Corrosion degree of reinforcement obtained from mass loss measurements.

The results for the degree of reinforcement corrosion obtained from mass loss measurements through Equation (1) are illustrated in Figure 5. As can be seen, generally, the transverse stirrups yielded more severe corrosion than the longitudinal rebar under identical impressed current and duration. This may be caused by the comparatively shorter distance of ties to the surface of concrete, which leads to a possible higher concentration of chloride ions [46]. Additionally, the diameter of longitudinal rebar (i.e., 10 mm in this study) was greater than that of the ties (i.e., 6 mm in this study). In this regard, the mass loss ratio of transverse stirrups with smaller diameter was higher than that of the longitudinal rebar, according to Faraday’s second Law of Electrolysis [47].

**Figure 6:**
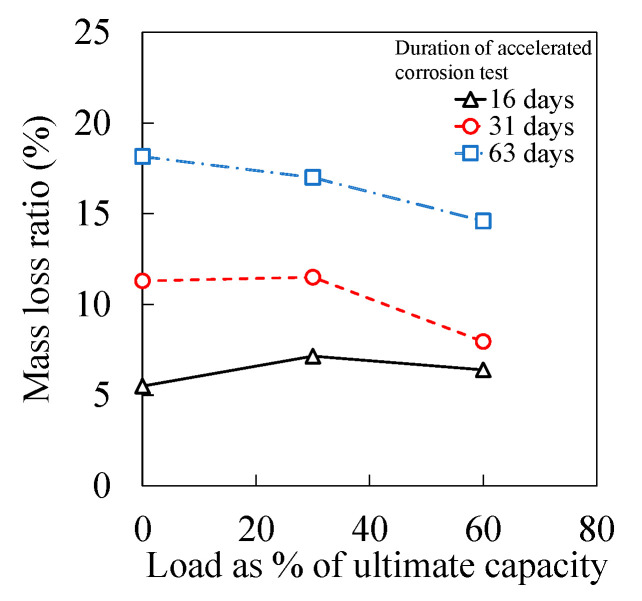
Sustained load versus mass loss ratio under different durations of accelerated corrosion test.

Figure 6 presents the results of mass loss ratio in the cases of different magnitudes of sustained axial load. The mass loss ratio in Figure 6 was the average value of longitudinal rebar and transverse stirrups shown in Figure 5. Generally, under identical duration of accelerated corrosion test, the degree of reinforcement corrosion was inversely proportional to the magnitude of sustained axial load. The compressive stress induced by the sustained axial load changed the porosity of concrete, reducing the chloride diffusion coefficient of concrete [41,48,49,50,51]. Additionally, steel subjected to compressive stress exhibited better corrosion-resistant performance than that under no loading condition in a chloride-contaminated environment [38,39].

### 3.2. Cracking Maps

**Figure 7:**
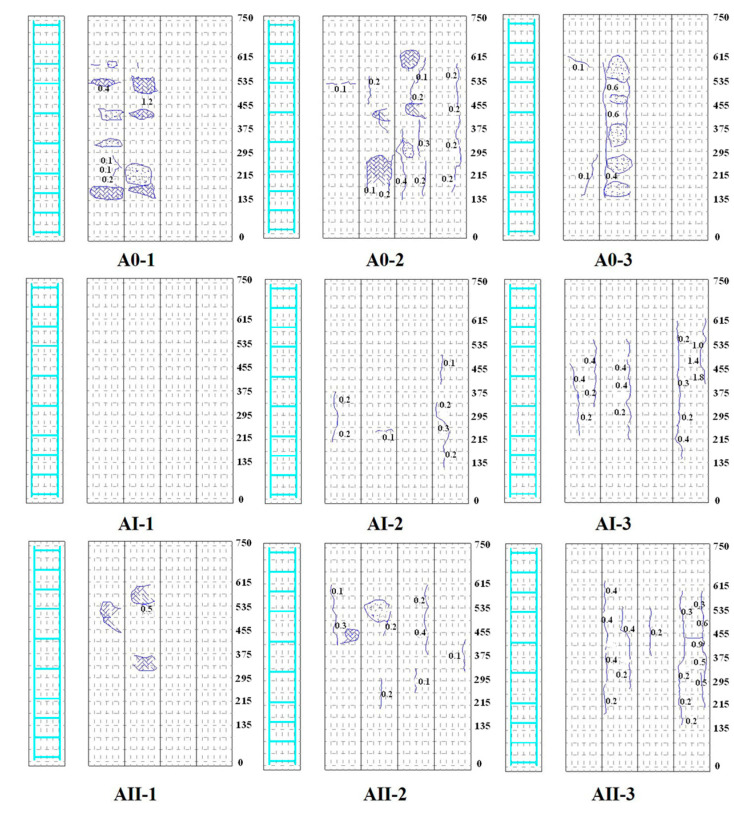
Cracking maps of RC column specimens. The details for the value of sustained load and duration of corrosion tests for specimens A0-1, A0-2, A0-3, AI-1, AI-2, AI-3, AII-1, AII-2, AII-3 are listed in Table 2.

Figure 7 presents the cracking maps of corroded RC columns, which were drawn after the corrosion test. Generally, as the degree of reinforcement corrosion increased, the number of cracks increased, as did the crack width [1]. In the case of corroded columns without sustained load, local bulge and spalling of concrete cover in the vicinity of transverse stirrups were observed, especially at a high degree of corrosion (e.g., A0-3). In the case of columns subjected to sustained load, less local bulge and spalling in the region of transverse stirrups was observed compared to those without sustained load. This may be due to the offset between the sustained compressive stress and the tensile stress induced by the pressure arising from corrosion-induced expansion of ties. On the other hand, fewer and wider longitudinal cracks along the main rebar were exhibited for specimens under sustained load, compared to those without sustained load at similar degree of corrosion. The reason could be the coupling effect of transversal expansion as a result of the Poisson effect and the tensile stress due to the corrosion-induced expansion of longitudinal rebar.

### 3.3. Failure Characteristics of Specimens

**Figure 8:**
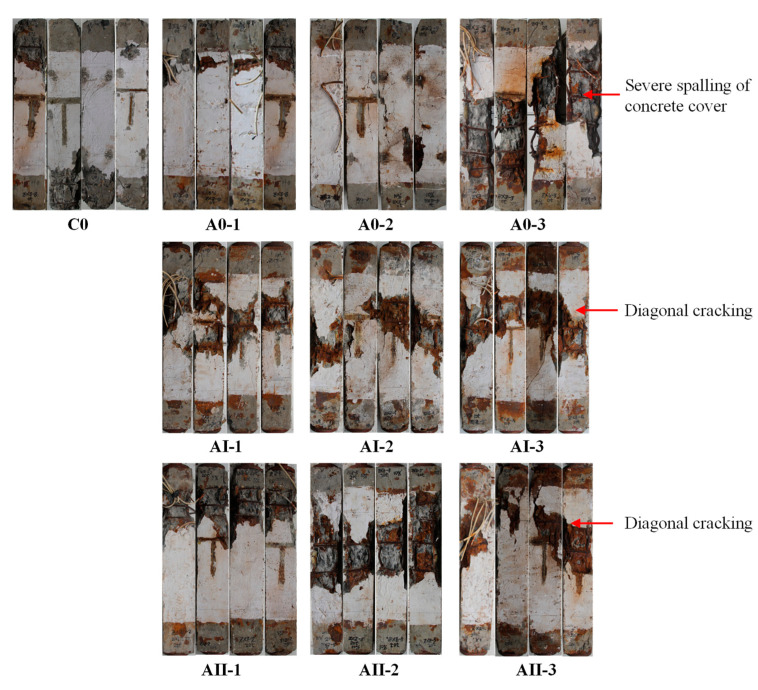
Failure characteristics of RC column specimens. The details for the value of sustained load and duration of corrosion tests for specimens C0, A0-1, A0-2, A0-3, AI-1, AI-2, AI-3, AII-1, AII-2, AII-3 are listed in Table 2.

After sustained loading and corrosion test, the sustained load was relived and column specimens were subjected to loading test with hydraulic pressing, as shown in Figure 4. The characteristics of failure for the column specimens, with and without simultaneous sustained concentric axial load and reinforcement corrosion, were demonstrated in Figure 8. As can be seen from Figure 8, irrespective of different degrees of reinforcement corrosion or sustained loading conditions, the failure of RC columns was basically due to the spalling of the concrete cover around the mid-height, followed by the buckling of the main rebar [52,53,54].

With respect to the influence of reinforcement corrosion, as the degree of corrosion increased, as in the case of A0-3 shown in Figure 8, severe spalling of concrete cover was observed. On the other hand, in cases with a high degree of corrosion (i.e., A0-3, AⅠ-3, AⅡ-3), diagonal cracking with an angle around 40° occurred, indicating that the failure of RC columns tended to be brittle.

There was no considerable effect of sustained axial load on the failure of RC columns. As Figure 6 demonstrated, in the case of the 63-day corrosion test, as the magnitude of sustained load increased from 0% to 60%, the mass loss ratio of reinforcement decreased from 18% to 15%. Such an amount of reduction in the corrosion degree may not be significant to affect the failure of RC columns.

### 3.4. Behavior of Specimens

**Figure 9:**
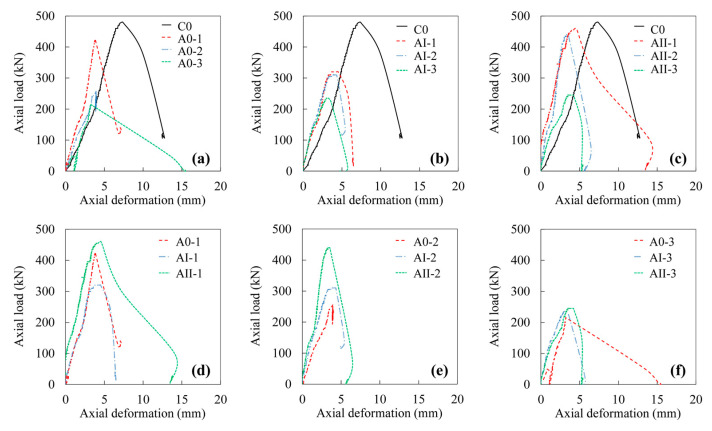
Axial load–axial deformation responses of column specimens. (**a**) Unloaded specimens with different corrosion degrees, (**b**) Specimens subjected to 30% ultimate load and various corrosion degrees, (**c**) Specimens subjected 60% ultimate load and various corrosion degrees, (**d**) Specimens subjected 16-days accelerated corrosion and various sustained load, (**e**) Specimens subjected to 31-days accelerated corrosion and various sustained load, (**f**) Specimens subjected to 63-days accelerated corrosion and various sustained load.

Figure 9 presents the axial load–axial deformation responses of RC column specimens which were subjected to different corrosion levels and magnitudes of sustained loads. The results of the specimens tested were summarized in Table 3. The ductility (*D*) of the column specimens was determined by Equation (2) [52,53,55]:

(2) $$D=\frac{\xi_{0.85}}{\xi_{y}},$$

where *ξ*_0.85_ is the axial deformation corresponded to the 85% of the ultimate axial load in the descending part of the axial load–axial deformation response, and *ξ*_y_ is the yield axial deformation which is determined as the axial deformation corresponded to the intersection point of two lines. One is the extension line between the origin and the point representing 75% of the ultimate axial load in the ascending part of the axial load–axial deformation response. The other line is the horizontal line passed through the ultimate axial load.

The effects of reinforcement corrosion are indicated in Figure 9a–c. As the degree of corrosion increased, the ultimate axial load as well as the corresponding axial deformation decreased. Similar trends were observed for specimens with and without sustained loads. The ductility of the columns, generally, was inversely proportional to the degree of corrosion, as shown in Table 3. The more severe the reinforcement corrosion, the more brittle the columns. This result agreed with the observations from the failure pattern of RC columns.

With respect to the influences of sustained load, the highest ultimate axial load was observed in the case of specimens subjected to the highest sustained load (i.e., 60% of ultimate capacity in this study), as can be seen in Figure 9d–f. Nevertheless, the ultimate axial load was not monotonically increased with increasing the sustained load in all cases. This may be due to the dissimilarities in the mass loss of corroded steels even with identical duration of corrosion test, which could affect the ultimate load carrying capacity of RC columns as well.

Additionally, specimens subjected to simultaneous reinforcement corrosion and sustained loads yielded larger slopes of the linear ascending region in the axial load–axial deformation responses, indicating a higher stiffness compared to uncorroded specimens under no sustained load (i.e., C0).

**Figure 10:**
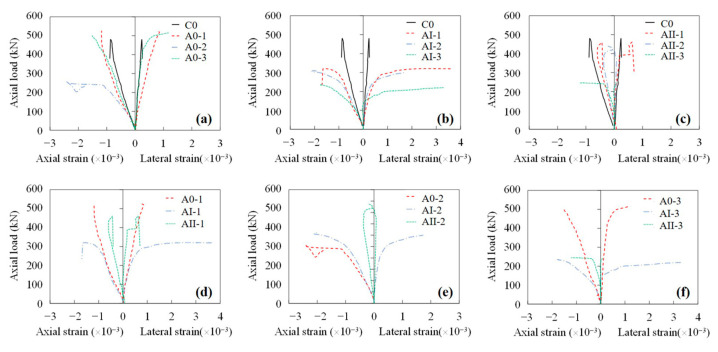
Axial load–axial strain and axial load–lateral strain responses of column specimens. (**a**) Unloaded specimens with different corrosion degrees, (**b**) Specimens subjected to 30% ultimate load and various corrosion degrees, (**c**) Specimens subjected 60% ultimate load and various corrosion degrees, (**d**) Specimens subjected 16-days accelerated corrosion and various sustained load, (**e**) Specimens subjected to 31-days accelerated corrosion and various sustained load, (**f**) Specimens subjected to 63-days accelerated corrosion and various sustained load.

Figure 10 illustrated the axial load–axial strain and the axial load–lateral strain responses of the specimens. The axial strain was greater than the lateral strain, resulting in a Poisson’s ratio of ~0.3. As the corrosion level increased, for unloaded specimens and specimens subjected to a load level of 30%, larger axial strain and lateral strain were observed, whereas for specimens subjected to a load level of 60% there was no considerable influence of corrosion degree on the strain of column specimens, as illustrated in Figure 10c. In terms of the sustained load, as the magnitude of sustained load increased, the axial strain as well as the lateral strain decreased, as presented in Figure 10d–f.

## 4. Conclusions

This study investigated mechanical performance of reinforced concrete (RC) columns subjected to simultaneous reinforcement corrosion and sustained concentric axial load.

Under an identical duration of an accelerated corrosion test, transverse stirrups yielded a higher degree of corrosion than that of the main rebar. Local bulge and spalling of concrete cover in the vicinity of transverse stirrups were observed in unloaded RC column specimens. In the case of column specimens subjected to sustained load, longitudinal cracks along the main rebar instead of local bulge in the vicinity of stirrups were more exhibited. The average corrosion degree of longitudinal rebar and transverse stirrups were generally decreased as the magnitude of sustained axial load increased.

Reinforcement corrosion led to the decrease of ultimate axial load and the increase of both axial and later strain of unloaded RC columns. In contrast, for column specimens subjected to different sustained loads under similar degree of corrosion, the highest value of ultimate axial load as well as the smallest axial and lateral strains were exhibited in specimens subjected to the greatest sustained load (i.e., 60% of ultimate capacity in this study).

RC columns subjected to simultaneous reinforcement corrosion and sustained axial load exhibited better performance with respect to the degree of reinforcement corrosion, stiffness, and ultimate axial load compared to unloaded columns. However, uncorroded RC columns exhibited better performance in terms of the ultimate axial load and axial deformation, compared with corroded RC columns under both loaded and unloaded conditions. The coupled effect of reinforcement corrosion and sustained load should be considered in predicting the mechanical performance as well as the reliability analysis of RC columns.

## Figures and Tables

**Table 1:** Mixture proportion of RC column specimens.

Material	Content (kg/m^3^)
Type Ⅰ cement	191
Slag	191
Water	203
Fine aggregate	766
Coarse aggregate	1149

**Table 2:** Experimental program.

Column	Sustained Load as % of Ultimate Capacity	Duration of Accelerated Corrosion Test (d)
C0	0	0
A0-1	0	16
A0-2	0	31
A0-3	0	63
AⅠ-1	30	16
AⅠ-2	30	31
AⅠ-3	30	63
AⅡ-1	60	16
AⅡ-2	60	31
AⅡ-3	60	63

**Table 3:** Experimental results of column specimens.

Column	Ultimate Axial Load (kN)	Axial Deformation at Ultimate Axial Load (mm)	Ductility
C0	479.5	7.40	1.52
A0-1	425.5	3.82	1.07
A0-2	257.0	3.93	1.01
A0-3	215.5	3.20	1.34
AⅠ-1	320.0	4.34	1.61
AⅠ-2	310.0	4.11	1.51
AⅠ-3	235.0	3.24	1.48
AⅡ-1	460.0	4.42	2.63
AⅡ-2	440.0	3.33	1.32
AⅡ-3	245.0	3.48	1.30

## Data Availability

Some or all data, models, or code that support the findings of this study are available from the corresponding author upon reasonable request.
